# Calorie restriction-induced leptin reduction and T-lymphocyte activation in blood and adipose tissue in men with overweight and obesity

**DOI:** 10.1038/s41366-024-01513-7

**Published:** 2024-03-27

**Authors:** Rebecca L. Travers, William V. Trim, Alexandre C. Motta, James A. Betts, Dylan Thompson

**Affiliations:** 1 Centre for Nutrition, Exercise and Metabolism (CNEM), Department for Health, University of Bath, Bath, BA2 7AY UK; 2 Unilever Food & Health Research Institute R&D, Vlaardingen, The Netherlands; 3 Present Address: Department of Systems Biology, Harvard Medical School, Boston, MA MA02115 USA; 4 Present Address: IMcoMET BV, Vlaardingen, The Netherlands

**Keywords:** Obesity, Metabolism

## Abstract

**Background:**

T-Lymphocyte activation is modulated by the adipokine leptin and serum concentrations of this hormone can be reduced with short-term calorie restriction. The aim of this study was to understand whether leptin per se is important in determining levels of T-lymphocyte activation in humans, by investigating whether the reduction in leptin concentration following calorie restriction is associated with a decrease in T-Lymphocyte activation in blood and adipose tissue.

**Methods:**

Twelve men with overweight and obesity (age 35–55 years, waist circumference 95–115 cm) reduced their calorie intake by 50% for 3 consecutive days. Blood and subcutaneous adipose tissue were obtained for isolation of immune cells and cytokine analysis. CD4+ and CD8 + T-Lymphocytes were identified and characterised according to their expression of activation markers CD25 and CD69 by flow cytometry.

**Results:**

Serum leptin was reduced by (mean ± SEM) 31 ± 16% (*p* < 0.001) following calorie restriction. The percentage of blood CD4 + CD25 + T-lymphocytes and level of CD25 expression on these lymphocytes were significantly reduced by 8 ± 10% (*p* = 0.016) and 8 ± 4% (*p* = 0.058), respectively. After calorie restriction, ex vivo leptin secretion from abdominal subcutaneous adipose tissue explants was not changed, and this corresponded with a lack of change in adipose tissue resident T-Lymphocyte activation.

**Conclusions:**

Serum leptin was reduced after calorie restriction and this was temporally associated with a reduction in activation of blood CD4 + CD25 + T-Lymphocytes. In abdominal subcutaneous adipose tissue, however, leptin secretion was unaltered, and there were no observed changes in adipose resident T-Lymphocyte activation.

## Introduction

Leptin is a cytokine-like hormone primarily produced by adipocytes in proportion to body fat mass with blood concentrations typically reflecting energy stored in adipose tissue [1]. Leptin acts centrally on the hypothalamus to regulate energy intake and expenditure but also influences a variety of peripheral physiological functions, including immune system function [2].

The leptin receptor (Ob-R) is widely-expressed across the immune system, including neutrophils, monocytes, macrophages, T-lymphocytes, B-lymphocytes, mast cells, dendritic cells and NK cells [2]. Leptin elicits dose-dependent effects on T-lymphocyte expression of the ‘early’ activation marker CD69 and ‘late’ activation maker CD25 in vitro (with co-stimulation from PHA or Con A), after 12 h and 48 h, respectively [3]. In humans, leptin is predominantly produced by subcutaneous adipose tissue due to its relative size and rate of production from this depot compared to other adipose tissues [4, 5]. Adipose tissue resident CD4+ and CD8 + T-lymphocytes show greater levels of activation (levels of CD69 and CD25 expression) with increasing adiposity and we proposed that activation may be related to heightened adipose tissue secretion of leptin [6]. As well as driving T-lymphocyte activation, leptin also promotes T-lymphocyte proliferation/ survival, in vitro [7, 8]. Whether leptin per se is a key factor in determining the levels of T-lymphocyte activation in human adipose tissue is unclear.

Leptin can be dramatically reduced with short periods of ‘severe’ calorie restriction [9–12]. Furthermore, expression of T-lymphocyte activation markers CD69 and CD25 also respond rapidly to mitogen stimulation, with changes being detectable after just a few hours [3, 13]. Thus, a short period of severe calorie restriction and reduction in leptin secretion may provide a route to test whether T-lymphocyte activation in adipose tissue and blood can be modified in-vivo in humans.

The aim of this research was therefore to investigate whether a calorie restriction-mediated reduction in leptin is temporally associated with reduced T-lymphocyte activation within blood and adipose tissue.

## Materials and methods

### Experimental design

Twelve men with overweight/obesity aged between 35–55 years were recruited from the local community following ethical approval from the South West, Frenchay NHS Research Ethics Committee (REC Reference: 12/SW/0324). Each participant gave written informed consent. Only participants with overweight and obesity with a waist circumference >94 cm [14] were recruited since these individuals showed greatest activation of T-lymphocytes within adipose tissue in our prior work [6]. After a 1-week period of monitoring energy intake and expenditure to confirm ‘energy balance’, participants reduced their caloric intake to 50% of their normal intake for 3 consecutive days (Supplementary Table 1). Participants attended the Physiology Laboratory at the University of Bath before and after this intervention for analysis of immune cell activation and markers of inflammation and metabolism in blood and adipose tissue. An oral glucose tolerance test was also performed to assess glycaemic control before and after the 3-day calorie restriction intervention. Individuals were excluded from participation if they smoked, had personal history of cardiovascular disease, metabolic disease or dyslipidaemia, or were taking medications that may influence lipid or carbohydrate metabolism or immune system function. It was also required that participants had been weight stable for more than 3 months (no change in weight +/−3%) [15]. This study is registered at Clinicaltrials.gov (Reference: NCT02473835).

### Sample size determination

There are no data regarding changes in T-lymphocyte activation in human adipose tissue in response to calorie restriction. However, significant differences in T-lymphocyte activation have been observed between lean and individuals with obesity [6]. A short period of calorie restriction can reduce serum leptin values by around 40% [9], which is sufficient to reduce typical values for a person with obesity to those of a lean person. Thus, a similar reduction in T-lymphocyte activation in response to calorie restriction might reasonably be anticipated. Previous data indicate that the CD69 mean fluorescence intensity (MFI) for lean CD4 + CD69+ cells is 288 (+/45 SD) and in individuals with obesity is 411 (+/31 SD) with an effect size of 3.08 (G-Power) [6]. To account for potentially greater variability in other activation markers, we recruited 12 participants.

### Monitoring of energy balance

Participants were weighed before and after a 1-week period of ‘energy balance monitoring’ to ensure weight stability using a digital balance (Tanita Corp.; Amsterdam, Netherlands). Participants were fitted with a combined heart rate and accelerometry monitor (Actiheart™; Cambridgeshire, UK) to determine habitual total energy expenditure (TEE) [16]. TEE was adjusted for measured resting metabolic rate (RMR) which was measured by indirect calorimetry [17]. Thus, TEE was calculated as the product of recorded estimates of activity energy expenditure + RMR + diet induced thermogenesis (estimated at 10% of TEE; [18]). Physical activity level (PAL) was then estimated by dividing TEE with basal metabolic rate [19] (here taken as RMR measured in the morning following an overnight fast). A weighed food and fluid intake record was used during this period to estimate participants’ energy intake with dietary analysis performed using COMP-EAT Pro software (v.5.8.0, Nutrition Systems; UK). Participants were asked not to make any conscious changes to their normal lifestyle habits/routines during this period and, to avoid influencing their habitual routines, were not told that the activity monitoring and food records would be used to directly influence the diet prescribed/given during the 3-day calorie reduction period. This analysis was then used to confirm that participants were in a state of energy balance and to write a diet prescription for the 3-day intervention. The aim was to ensure that participants received 50% of their ‘normal’ calorie requirements (i.e., average of energy intake and energy expenditure) using foods they would normally consume. Since there are errors associated with estimations of total energy expenditure [16] and recording dietary intake (in particular underreporting) [20], we stipulated a priori that total energy intake and energy expenditure values had to be within 25% of each other during the energy balance assessment and, furthermore, that the prescribed calorie intake had to be within 40–60% of both the total energy expenditure and dietary intake values. If either of these requirements were not met, participants were asked to repeat the monitoring phase. A summary of dietary calculations is available in Supplementary Fig. 1.

### Calorie restriction protocol

To determine the exact calorie intake required to achieve a calorie restriction of 50% normal energy requirements for the 3-days, an average of energy expenditure and dietary intake from the monitoring period was taken. This value was then divided in half to give the ‘target’ calorie value for each of the 3-days in the diet. Subsequently, three separate days from the participant’s one-week diet record were selected and the weight of each item adjusted to meet this daily target kcal value whilst maintaining the overall relative proportions so that participants’ typical diet composition remained unaltered. A worked example showing how the weights of food items were adjusted whilst maintaining overall proportions is shown in Supplementary Fig. 1. During the 3-day intervention period, participants were asked to record the timing of each food/fluid intake within the prescribed food diary and to confirm that they had consumed the correct amount of food to help improve compliance with diet instructions. Participants were asked not to make any conscious changes to habitual physical activity during the calorie restriction period.

### Sample collection days

Participants were asked not to perform any strenuous physical activity for 48 h and to refrain from consuming caffeine/alcohol for 24 h before both trial days (i.e., pre- and post-intervention). Trial days were scheduled so participants had been free from any self-reported illness for a minimum of 2 weeks in order to reduce immune system disturbance. On both main trial days, participants arrived at the Physiology Resting Laboratory in the morning following a 10 h fast (approximately 8 am) and after consuming 1 pint of water upon waking. Participants arrived in the laboratory at the same time on both trial days. Measurements of height, waist circumference and body mass (post-void using a digital balance; TANITA corp.) were determined on both trial days. Participants’ body composition was characterised at baseline using dual energy X-ray absorptiometry (DEXA; Discovery, Hologic; Bedford, UK) and estimates of total and central fat mass [L1-L4; [21]] and fat mass index (FMI; kg/m^2^) [22] determined.

#### Blood and adipose sampling

A cannula was inserted into an antecubital forearm vein and blood sample(s) taken for isolation of peripheral blood mononuclear cells (PBMCs) by density gradient separation (Lympholyte; Cedarlane Laboratories Ltd.; Ontario, Canada) and analysis of plasma and serum metabolic/inflammatory markers [6]. Subcutaneous adipose tissue samples (~1 g) were obtained under local anaesthetic (1% lidocaine) approximately 5 cm lateral to the umbilicus using a ‘needle aspiration’ technique [23]. Approximately 100 mg whole adipose tissue was transferred to an RNase/DNase free sterile centrifuge tube, homogenised in Trizol reagent (Invitrogen; MA, USA) and frozen on dry ice for later RNA isolation. The remainder was used for adipose tissue culture [explants cultured for 3 h at a concentration of 100 mg/ mL [4]], and preparation of the stromavascular fraction (SVF), both described previously [6, 24]. Due to the limited size of some adipose tissue samples, priority was given to preparing tissue for analysis of SVF to address the main aim of this study (*n* = 12). Lowest priority was given to gene expression analysis. Paired samples (pre- and post-calorie restriction) were available for 9 participants for explant secretion analysis and 7 participants for gene expression analysis.

#### Oral glucose tolerance test

Participants were asked to consume a glucose drink consisting of 75 g anhydrous glucose (maltodextrin) solution (Polycal, Nutricia; Wiltshire, UK) and cannula blood samples were taken every 15 min for the following 2 h for measurement of plasma glucose and serum insulin concentrations.

### Analysis of SVF and PBMCs by flow cytometry

Flow cytometry (using the FACSverse, BD; NJ, USA) was used to identify CD4+/CD8 + T-lymphocytes (CD45+ CD3+ cells) in SVF and PBMCs together with respective levels of activation. Due to the limited size of the SVF samples for analysis, cells were labelled using a single antibody cocktail comprising; CD4-FITC, CD8-PE-Cy7, CD69-APC, CD25-APC-Cy7, CD3-V450 and CD45-V500 (BD; USA). PBMCs were labelled using; CD45-V500, CD3-V450, CD295-FITC, CD220-PE, CD4-PerCP, CD8-PE-Cy7, CD69-APC and CD25-APC-Cy7 (BD; USA). To assess the impact of calorie restriction on T-cell activation, first CD45 + CD3 + T-cells were identified in all samples, which were then split into either CD4+ or CD8 + T-cell subsets, respectively, to identify helper and cytotoxic T-cells. From here, the proportion of each of these T-cell subsets that was expressing either CD69 or CD25—early and late T-cell activation markers, respectively—was quantified for our analysis of the amount of activated T-cells present. Moreover, the degree of activation, on a per-cell basis, was also assessed on these helper (CD4+) and cytotoxic (CD8+) T-cells by measuring the amount of either CD25 or CD69 present on each cell, expressed as median fluorescence intensity (MFI). This method on cell surface protein expression density was also applied to both the leptin (CD295) and insulin (CD220) receptors on T-cells in PBMCs.

### RT-PCR

Total RNA was extracted from whole adipose tissue, quantified and 1 μg reverse transcribed to cDNA as described previously [6]. Real-time PCR was performed using a StepOne^TM^ (Applied Biosystems; MA, USA) with pre-designed primers and probes obtained from Applied Biosystems for measurement of *LEPTIN* (Hs00174877_m1), *ADIPONECTIN* (Hs00605917_m1), *GLUT4* (Hs00168966_m1), IRS2 (Hs00275843_s1), *HSL* (Hs00193510_m1), *LPL* (Hs01012567_m1), *PPARγ* (Hs01115513_m1), *MCP1* (Hs00234140_m1), *IL6* (Hs00985639_m1), *IL8* (Hs99999034_m1), *IL1Rα* (Hs00893626_m1), and *IL18* (Hs00155517_m1) expression. Peptidylpropyl isomerase A (*PPIA*) was used as an endogenous control [25]. Results were analysed using the comparative Ct method and expression normalised to an internal calibrator specific to each gene using the formula 2^−∆∆C^_T_; where ∆∆C_T_ is [(C_T_ gene of interest − C_T_ PPIA) − lowest ∆C_T_ for gene of interest] and statistical analysis performed on LN-transformed values [26].

### Biochemical analysis

Plasma glucose, serum total cholesterol, HDL cholesterol, triglycerides and CRP concentrations and ALT activity were measured using commercially available assay kits and analyser (Daytona Rx, Randox; Crumlin, UK). ELISA was used for the measurement of serum Insulin concentrations (Mercodia; Uppsala, Sweden), and both serum and adipose tissue Leptin and Adiponectin secretion (R&D systems; MN, USA). A fluorescent bead multiplex system (Luminex, BIO-RAD; CA, USA) was used for the measurement of serum and adipose tissue secretion of TNFα, IL-10, IL-8, MCP-1, IL-1a, IL-1Ra, IL-18, IL-6, G-CSF, IP-10, MIP-1β, and M-CSF. Serum IL-6, G-CSF, M-CSF, IL-1a, and IL-1Ra were detectable in fewer than 3 individuals so results were not included in statistical analysis. TNFα and IL-10 were not detectable in either serum or adipose culture supernatant.

### Statistical analysis

All data are presented as mean and standard deviation (SD) within the body of the text and tables, data within graphs are presented as mean values ± SEM with individual values overlaid, unless otherwise stated. Estimates of glucose control were calculated using homoeostasis model assessment for insulin resistance [HOMA-IR; [27]] and insulin sensitivity index [ISI comp/ Matsuda index; [28]]. LDL cholesterol was calculated using the Friedewald equation [29] Comparisons were made between pre- and post- calorie restriction values using two-tailed, paired *t*-tests, or non-parametric equivalents where data were non-normally distributed (Shapiro–Wilks *p* > 0.05). Correlation analysis was performed using Pearson’s *r*. Statistical analysis was performed using GraphPad Prism 9.1.2 (GraphPad Software, LLC.; FL, USA). Effect sizes were calculated using Cohen’s *d. p* ≤ 0.05 was considered to be statistically significant.

## Results

Energy intake was reduced from 2499 ± 412 kcal/ d to 1320 ± 183 kcal/ d during the 3-day calorie restriction period [50% of mean (energy intake and TEE) from energy balance monitoring week]. Specifically, energy intake during the calorie restriction period was 53.1 ± 3.9% of pre-intervention calorie intake, and 48.7 ± 6.0% of average total energy expenditure across the 3-day intervention. There was a significant reduction in body mass, BMI, waist circumference, plasma glucose concentration at 2 hr post-OGTT, plasma triglyceride concentration, and plasma ALT activity; with significant increases in calculated plasma LDL-Cholesterol concentration and plasma NEFA concentration after 3 days of 50% reduction in calorie intake (Table 1).

**Table 1:** Anthropometric measures and blood markers of metabolism before and after 3 days of 50% calorie restriction.

Physical characteristics	Pre-calorie restriction	Post-calorie restriction	Δ Change mean ± 95% CI	*p*
Body mass (kg)	93.1 ± 9.8	91.5 ± 9.7	−1.6 (−1.9 to −1.2)	<0.001
Body mass index (kg/m^2^)	29.0 ± 2.4	28.5 ± 2.3	−0.5 (−0.6 to −0.4)	<0.001
Waist circumference (cm)	104.6 ± 6.0	103.6 ± 6.1	−1.1 (−1.4 to −0.7)	<0.001
Height (m)	1.79 ± 0.10	ND	ND	
Fat mass (%)	26.9 ± 6.0	ND	ND	
Fat mass (kg)	26.5 ± 6.1	ND	ND	
L1–L4 fat mass (kg)	3.8 ± 1.0	ND	ND	
Fat mass index (kg/m^2^)	8.23 ± 1.66	ND	ND	
Resting metabolic rate (kcal/day)	1814 ± 117	ND	ND	
PAL	1.54 ± 0.21	ND	ND	

Blood biochemistry	Pre-calorie restriction	Post-calorie restriction	Δ Change mean ± 95% CI	*p*
Insulin – fasting (pmol/L)	46.7 ± 33.0	42.6 ± 31.6	−4.1 (−9.4 to 1.2)	0.758
Glucose – fasting (mmol/L)	5.1 ± 0.6	5.1 ± 0.4	−0.1 (−0.3 to 0.2)	0.623
Glucose at 2 h (mmol/L)	6.8 ± 1.6	6.0 ± 1.5	−0.8 (−1.3 to −0.4)	0.002
HOMA-IR	1.8 ± 1.3	1.6 ± 1.3	−0.2 (−0.4 to 0.1)	0.765
Matsuda-ISI^a^	6.21 ± 5.75	7.29 ± 6.19	1.08 (0.20 to 1.96)	0.674
ALT (U/L)	40.2 ± 20.6	34.9 ± 17.5	−5.3 (−10.6 to 0.0)	0.051
Triglycerides (mmol/L)	1.36 ± 0.67	1.05 ± 0.56	−0.31 (−0.51 to −0.11)	0.005
NEFA (mmol/L)	0.30 ± 0.11	0.58 ± 0.16	0.27 (0.22 to 0.33)	<0.001
Total-Cholesterol (mmol/L)	5.3 ± 0.7	5.6 ± 0.8	0.3 (0.0 to 0.5)	0.046
LDL-Cholesterol (mmol/L)	3.47 ± 0.58	3.86 ± 0.73	0.39 (0.10 to 0.68)	0.013
HDL-Cholesterol (mmol/L)	1.19 ± 0.17	1.22 ± 0.18	0.02 (−0.08 to 0.13)	0.741

Mean ± SD shown for pre and post values, with mean ± 95% confidence intervals used to express delta change scores. Differences between the two days were analysed by two-tailed, paired, Student’s *t* tests or non-parametric equivalent where data were non-normally distribution (Shapiro–Wilk’s *p* > 0.05), *p*-values shown.

*CI* confidence intervals, *L1-L4* central fat estimated between lumbar regions L1-L4 using DEXA, *PAL* physical activity level (total energy expenditure/basal metabolic rate), *ALT* alanine transaminase, *HDL* high-density lipoprotein, *HOMA-IR* homoeostasis model assessment for insulin resistance, *LDL* low-density lipoprotein, *ND* not determined, *NEFA* non-esterified fatty acids.

^a^T-AUC for insulin and glucose and Matsuda-ISI represent *n* = 11 due to incomplete data. All blood measures are fasting samples unless otherwise stated.

### Changes in leptin and immune cell activation in blood and adipose tissue

**Fig. 1:**
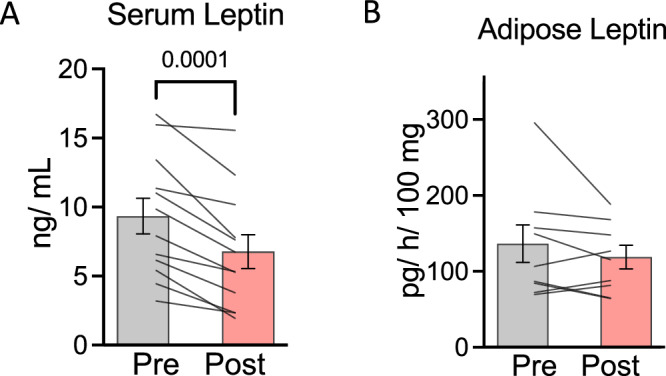
Serum and adipose tissue leptin production before and after 3 days of 50% calorie restriction. **A** Serum leptin concentrations before and after 3 days of 50% calorie restriction. Data are presented as group mean ± SEM with individual responses overlaid. Data represent *n* = 12. **B** Leptin secretion by whole adipose tissue explants cultured for 3 h before and after 3 days of 50% calorie restriction. Data are presented as group mean ± SEM with individual responses overlaid. Data represent *n* = 8. Data were analysed using paired t-tests, or non-parametric equivalents where data were non-normally distributed (Shapiro–Wilks *p* > 0.05).

After 3 days of 50% calorie restriction, there was a significant reduction in serum leptin concentration of 31% from baseline (range 3% to 64%; Fig. 1A). Conversely, there was no statistically significant difference in leptin adipose tissue explant secretion ex vivo (Fig. 1B). There was also no relationship between the percent change in serum and adipose tissue ex vivo leptin concentration/ production (*r* = −0.265, *p* = 0.490; *data not shown*).

**Fig. 2:**
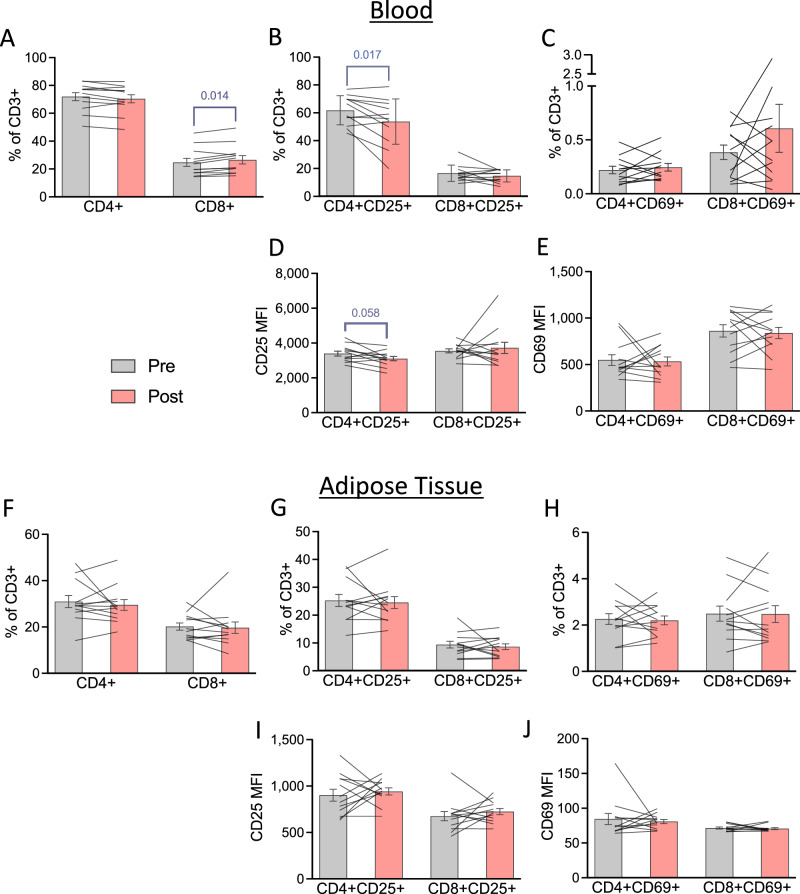
Changes in peripheral blood mononuclear cell and adipose tissue T-lymphocyte abundance and activation before and after 3 days of 50% calorie restriction. **A** Relative proportions of CD4+ and CD8+ events within total CD3+ events before and after 3 days of 50% calorie restriction in PBMCs. **B** Relative proportions of CD4+ CD25+ and CD8+ CD25+ events within total CD3+ events before and after 3 days of 50% calorie restriction in PBMCs. **C** Relative proportions of CD4+ CD69+ and CD8+ CD69+ events within total CD3+ events before and after 3-days of 50% calorie restriction in PBMCs. **D** CD25 MFI on CD4+ CD25+ and CD8+ CD25 + T-lymphocytes before and after 3-days of 50% calorie restriction in PBMCs. **E** CD69 MFI on CD4+ CD25+ and CD8+ CD25 + T-lymphocytes before and after 3-days of 50% calorie restriction in PBMCs. **F** Relative proportions of CD4+ and CD8+ events within total CD3+ events before and after 3-days of 50% calorie restriction in adipose tissue. **G** Relative proportions of CD4 + CD25+ and CD8 + CD25+ events within total CD3+ events before and after 3 days of 50% calorie restriction in adipose tissue. **H** Relative proportions of CD4 + CD69+ and CD8 + CD69+ events within total CD3+ events before and after 3-days of 50% calorie restriction in adipose tissue. **I** CD25 MFI on CD4+ CD25+ and CD8+ CD25+ T-lymphocytes before and after 3-days of 50% calorie restriction in adipose tissue. **J** CD69 MFI on CD4+ CD25+ and CD8+ CD25-+ T-lymphocytes before and after 3-days of 50% calorie restriction in adipose tissue. Data are presented as group means ± SEM at each time point with individual responses overlaid. All data represent *n* = 12. Data were analysed using paired t-tests, or non-parametric equivalents where data were non-normally distributed (Shapiro–Wilks *p* > 0.05). *MFI* median fluorescence intensity, *PBMCs* peripheral blood mononuclear cells.

In the PBMCs, CD8 + T-lymphocytes as a proportion of CD3+ cells were significantly elevated following calorie restriction (*p* = 0.014; Fig. 2A), whereas CD4+ cells were not different. Within the T-lymphocyte compartment, the proportions of CD4 + CD25+ cells as a proportion of CD3+ cells were significantly reduced following calorie restriction (*p* = 0.017; Fig. 2B). There was also a trend for a reduction in CD25 MFI on CD4 + CD25 + T-lymphocytes in PBMCs (*p* = 0.058, *d* = 0.63; Fig. 2D), whereas the expression of CD25 and CD69 on CD8 + T-lymphocytes and CD69 in CD4 + T-lymphocytes in PBMCs was not altered.

The expression of the leptin (CD295) and insulin (CD220) receptors was also measured on CD25+ /CD69+ CD4+ and CD8+ T-lymphocytes. The surface expression on a per-cell basis of CD220 on CD4+ CD25+ T-lymphocytes was significantly increased by calorie restriction (*p* = 0.009; Table 2). Moreover, the expression of CD220 on CD8+ CD25+ cells was increased with a large effect that approached statistical significance (*p* = 0.061, *d* = 0.83; Table 2).

**Table 2:** Peripheral blood T cell Leptin (LepR/CD295) and Insulin (InsR/CD220) receptor expression before and after 3 days of 50% calorie restriction.

	CD295 (LepR) MFI			CD220 (InsR) MFI		
Cell Type	Pre	Post	*p*	Pre	Post	*p*
CD4+ CD25+	230 ± 24	234 ± 27	0.627	86 ± 9	98 ± 17	0.009
CD4+ CD69+	6949 ± 8826	4589 ± 4677	0.425	1292 ± 1151	1323 ± 1226	0.954
CD8+ CD25+	269 ± 17	303 ± 75	0.155	123 ± 15	141 ± 27	0.061
CD8+ CD69+	1213 ± 211	1262 ± 392	0.635	566 ± 77	580 ± 72	0.644

Mean ± SD values shown and differences between the two days analysed by two-tailed, paired, Student’s *t* tests or non-parametric equivalent where data were non-normally distribution (Shapiro–Wilk’s *p* > 0.05). *n* = 12 for all, *p*-values shown.

*InsR* Insulin Receptor, *LepR* Leptin Receptor, *MFI* median fluorescence intensity.

### Inflammatory and metabolic changes in blood and adipose tissue

**Fig. 3:**
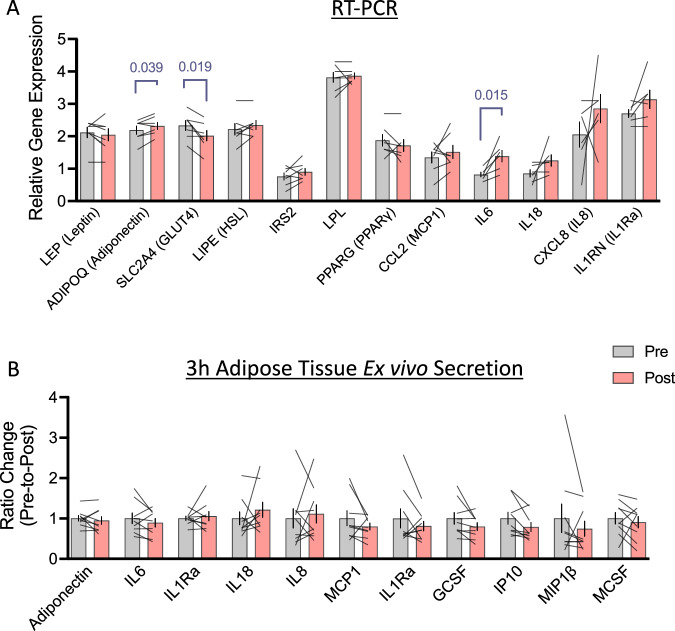
Gene expression and secretion changes in whole adipose tissue before and after 3-days of 50% calorie restriction. **A** Changes in relative gene expression of proteins related to metabolism and inflammatory cytokines within adipose tissue before and after 3-days of 50% calorie restriction. Data presented as the ratio change ± SEM with individual values overlaid of pre-to-post intervention of the raw mean 2^−∆∆Ct^. All represent *n* = 7, with the exception of *IL6, IL8, IL18, and IL1RA*, which represent *n* = 6. Gene names are presented with protein reference names in brackets, where necessary. **B** Adipokine and cytokine secretion by whole adipose tissue explants cultured for 3 h before and after 3-days of 50% calorie restriction. Data are presented as a ratio of the pre-to-post change in group mean at each time point ± SEM with individual responses overlaid. All represent *n* = 9. Data were analysed using paired t-tests, or non-parametric equivalents where data were non-normally distributed (Shapiro–Wilks *p* > 0.05). GCSF granulocyte-colony stimulating factor, IL interleukin, IP-10 inflammatory protein 10, MCP-1 monocyte chemotactic protein 1, MCSF macrophage colony-stimulating factor, MIP-1β macrophage inflammatory protein 1β, RT-PCR reverse transcription-polymerase chain reaction.

Adiponectin and a number of inflammatory cytokines were measured in the blood plasma before and after the 3-days of 50% calorie restriction. Of those that were detected (Table 3), plasma IL-18 concentration showed significant reductions after calorie restriction. Changes in a selection of genes related to inflammation and glucose/lipid metabolism in adipose tissue were also investigated. Both adiponectin and *IL6* mRNA showed a significant modest increase and *GLUT4* mRNA showed a reduction in expression within adipose tissue 3-days after 50% calorie restriction (*p* = 0.039, 0.015, and 0.019, respectively; Fig. 3A). Conversely, there were no significant changes in secretion of any of the proteins measured from whole adipose tissue explants following calorie restriction (Fig. 3B).

**Table 3:** Plasma adiponectin and markers of inflammation before and after 3-days of 50% calorie restriction.

Plasma protein	Pre-calorie restriction	Post-calorie restriction	Δ Change mean ± 95% CI	*p*
Adiponectin (µg/mL)	6.65 ± 2.70	6.39 ± 2.39	−0.26 (−0.38 to −0.14)	0.206
CRP (mg/L)	1.54 ± 1.51	1.51 ± 1.09	−0.04 (−0.78 to 0.70)	0.914
IL-6 (pg/mL)	0.67 ± 0.38	0.75 ± 0.38	0.08 (0.06 to 0.10)	0.606
IL-8 (pg/mL)	5.7 ± 2.9	5.5 ± 2.4	−7.3 (−10.9 to −3.7)	0.838
MIP-1β (pg/mL)	69.6 ± 27.4	72.1 ± 29.1	2.5 (−2.2 to 7.2)	0.261
IL-18 (pg/mL)	95.8 ± 41.3	88.4 ± 41.0	−0.2 (−1.0 to 0.5)	0.001
IP-10 (pg/mL)	347.0 ± 192.2	340.3 ± 213.2	−6.7 (−49.5 to 36.1)	0.936
MCP-1 (pg/mL)	49.8 ± 21.7	43.4 ± 18.2	−6.4 (−11.4 to −1.4)	0.441

Mean ± SD shown for pre and post values, with mean ± 95% confidence intervals used to express delta change scores. Differences between the two days were analysed by two-tailed, paired, Student’s *t* tests or non-parametric equivalent where data were non-normally distribution (Shapiro–Wilk’s *p* > 0.05). *n* = 12 for all, *p*-values shown.

*CI* confidence intervals, *CRP* C-reactive protein, *IP-10* inflammatory protein 10, *MCP-1* monocyte chemotactic protein 1, *MIP-1β* macrophage inflammatory protein 1β.

### Associations between leptin responses to calorie restriction and T-lymphocyte phenotypes

**Fig. 4:**
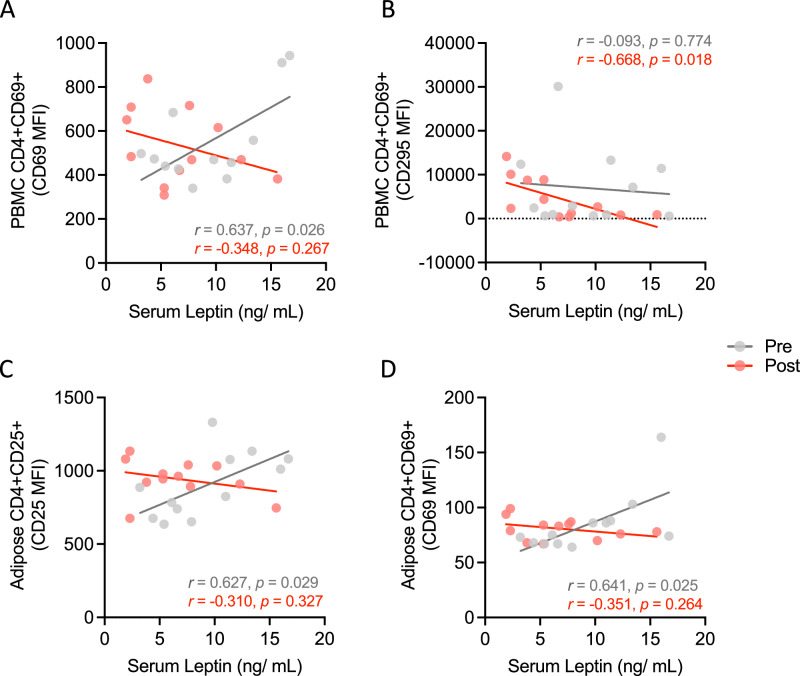
Associations between Serum leptin concentrations and markers of T-lymphocyte activation and leptin receptor expression before and after 3-days’ calorie restriction. **A** Association between PBMC derived CD4+ CD69 + T-lymphocyte CD69 MFI with serum leptin pre- and post-3 days of 50% calorie restriction. **B** Association between PBMC derived CD4+ CD69+ T-lymphocyte leptin receptor (CD295) MFI with serum leptin pre- and post-3 days of 50% calorie restriction. **C** Association between adipose tissue derived CD4+ CD25+ T-lymphocyte CD25 MFI with serum leptin pre- and post-3 days of 50% calorie restriction. **D** Association between adipose tissue derived CD4+ CD69+ T-lymphocyte CD69 MFI with serum leptin pre- and post-3 days of 50% calorie restriction. Correlations were performed using Pearson’s *r*. All represent *n* = 12. MFI median fluorescence intensity, PBMC peripheral blood mononuclear cells.

To understand the potential ramifications of altered circulating leptin on T-lymphocyte activation and leptin receptor expression following 3-days of 50% calorie restriction, we correlated (Pearson’s *r*) the absolute concentrations of serum leptin with T-lymphocyte activation marker expression (CD25/CD69 MFI) and leptin/insulin receptor expression. Statistically significant associations are presented in Fig. 4A–D. Of note, before the intervention, expression of the early T-lymphocyte activation marker CD69 on PBMC-derived CD4+ CD69+ cells (on a per-cell basis) was significantly related to serum leptin concentration, which was lost post-intervention (*r* = 0.637, *p* = 0.026 and *r* = −0.348, *p* = 0.267, respectively; Fig. 4A). However, the expression of the leptin receptor on these same cells was not statistically significant pre-intervention but was significantly negatively associated post-intervention (*r* = −0.093, *p* = 0.774 and *r* = −0.668, *p* = 0.018, respectively; Fig. 4B). Further, in adipose tissue CD4+ T-lymphocytes, the expression of CD25 on a per-cell basis was similarly (to PBMCs) significantly associated pre-intervention but was lost post- (*r* = 0.627, *p* = 0.029 and *r* = −0.310, *p* = 0.327, respectively; Fig. 4C), a pattern also apparent in the expression of CD69 on a per-cell basis in adipose tissue (*r* = 0.641, *p* = 0.025 and *r* = −0.351, *p* = 0.264, respectively; Fig. 4D).

## Discussion

In this study, we found that following 3-days of 50% calorie restriction, leptin concentrations in blood were lower and this was temporally associated with a reduction in the proportion and levels of activation of blood CD4+ CD25+ T-lymphocytes. In contrast, leptin secretion and gene expression in abdominal subcutaneous adipose tissue were not reduced and there was also no change in adipose tissue resident T-lymphocyte activation.

### Changes in T-lymphocyte activation in blood following acute calorie restriction

We show a positive association between ‘early’ (CD69) and ‘late’ (CD25) markers of CD4+ T-lymphocyte activation in both blood and adipose tissue with serum leptin concentrations. Previous in vitro studies have reported dose-dependent effects of leptin on CD4+ T-lymphocyte activation markers [3, 30, 31]. Notably, our observations were in the absence of any association between leptin receptor abundance on these cells pre-intervention with serum leptin concentrations.

Mechanistic studies have shown that, in response to strong mitogen stimulation, T-lymphocyte CD69 and CD25 expression are upregulated in vitro within 12 and 24–48 h, respectively [3, 32]. Similarly, mitogen activation of T-lymphocytes leads to an increase in leptin receptor expression on T-lymphocytes in mice and humans [33]. Collectively, these results suggest that, for leptin to exhibit an effect on T-lymphocyte activation and leptin receptor expression, dual stimulation by mitogen in the presence of elevated circulating leptin may be required. Thus, leptin alone may not (acutely) modulate T-lymphocyte activation or leptin receptor expression, though it cannot be ascertained from the current study whether leptin alone is a sufficient signal to impact T-lymphocyte activation over longer periods (weeks/months). It is also possible that leptin is important for T-lymphocyte signalling when energy is available to meet the demands for an immune response, which may not have been the case when energy intake is below requirements. Indeed, leptin deficiency/starvation appears to be associated with immunosuppression via an increase in T-regulatory cells [30, 34] and high levels of leptin as seen in individuals with obesity are implicated in the chronic low-grade inflammation observed via an increase in Th1 T-lymphocytes [35]. The present findings may, therefore, have relevance to our understanding of how T-lymphocyte activation is modulated by exposure to leptin in vivo. Indeed, there is the potential that elevated leptin concentrations in individuals with overweight and obesity during infectious cycles (e.g., seasonal flu) may potentiate hyper-inflammatory T-lymphocyte responses, and their chronic activation. If this is true, it would add to our understanding of why obesity may represent such a notable risk factor in infections such as influenza and SARS-CoV2 [36–38]. These findings may help explain prior reports of compromised immune function and heightened susceptibility to infection during rapid weight loss [39, 40].

### Differing leptin responses in blood and adipose tissue after acute calorie restriction

Despite changes in leptin and T-lymphocyte activation in the blood following calorie restriction, there were no corresponding changes in adipose tissue leptin production or T-lymphocyte activation. It is possible that, since adipose tissue is the primary site of leptin secretion, the adipose resident T-lymphocytes may not experience such a dramatic reduction in leptin exposure as in blood, or extracellular/interstitial leptin concentrations may remain above a threshold to maintain T-lymphocyte activation in adipose. Leptin is known to promote T-lymphocyte survival/proliferation, in vitro [7, 8], and CD25—the IL2RA chain—is also intimately associated with T-lymphocyte proliferation/survival, which may represent a potential overlapping mechanism between leptin and T-lymphocyte CD25/ IL-2 signalling-mediated proliferation. Indeed, such an overlap between these systems has been proposed, in vitro, wherein leptin primes CD4+ T-lymphocytes towards a Th1 lineage, stimulating IL2 and IFNγ production [3], which in-turn would activate T-lymphocytes via the IL2-receptor in an autocrine manner. However, future work should look to further delineate the CD4+ T-lymphocyte cluster, as leptin is also known to negatively regulate CD4+ CD25+ FoxP3+ regulatory T-lymphocytes [41], which may have influenced the present findings.

The lack of response in leptin gene expression and secretion in adipose tissue was an unexpected and intriguing finding. In-keeping with our results, it appears that in times of short term calorie restriction/starvation, despite a significant reduction in blood leptin concentrations, adipose tissue gene expression of leptin continues to reflect levels of adiposity [42, 43]. Indeed, much longer periods of calorie restriction (e.g. >3 weeks) and greater weight loss are required to elicit changes in leptin gene expression [44, 45]. In times of acute calorie restriction, the reduction in blood leptin serves as a signal to the brain and all systems in the body that there is an energy insufficiency irrespective of adiposity [46]. It is not clear where or how this reduction in blood leptin occurs. Our results suggest there is no change in the secretion of leptin from abdominal subcutaneous adipose tissue, so one or a combination of factors affecting half-life (clearance/degradation/binding of leptin to proteins in blood) or changes in secretion from other adipose depots or even non-adipose tissues [47] may be responsible for the reduced levels of leptin in blood.

### Considerations

It is important to highlight that the media used for the adipose tissue culture in our ex-vivo model was not supplemented with glucose/insulin or other potential stimuli of leptin secretion from adipocytes [48]. This was a deliberate decision to avoid stimulation of adipose tissue ex vivo, but it is possible that this may have produced different results to those had the adipose tissue remained in situ or in ex vivo conditions that recapitulated each individual’s in vivo environment. Future studies could therefore benefit from measuring arterio-venous differences to see if the total output from the adipose tissue is reduced with calorie restriction and whether there are depot-specific effects.

Several findings in the present study support the notion that a mild starvation-type response was induced following 3-days of 50% calorie restriction characterised by a profound reduction in triglycerides and an increase in NEFA seen in the blood. Adipose tissue gene expression of *GLUT4* was reduced following calorie restriction as occurs in human skeletal muscle during starvation [49] together with an increase in both *IL6* and adiponectin (*ADIPOQ)* gene expression. The potential implications of these changes in adipose tissue and whether these are seen with less ‘severe’ calorie restriction (as would be more typical of dieting for weight loss) certainly warrant further investigation.

### Limitations

It is important to note for purposes of wider relevance and interpretation of the present findings that this study was undertaken in a relatively small, homogeneous sample of males. Leptin exhibits a sexual dimorphism, with females exhibiting higher circulating leptin concentrations and greater rate of production by adipose tissues (independent of absolute adiposity), driven in-part by sex hormones and differential regional deposition of adiposity between males and females [50–53]. Finally, it is also known that there is a sexual dimorphism in the metabolic responses to short-term (<24 h) fasting [54], as well as considerable and wide-spread differences in the immune system of males and females, and across age groups [55–57]. Therefore, future studies should examine the role of calorie restriction in modulating circulating leptin concentrations and their potential influence on T-cell phenotypes in other populations.

This study was a pre-post observational study and not a randomised control trial. Thus, we cannot establish direct causation with this design. However, the effects of calorie restriction on leptin concentrations are unequivocal based on findings across multiple populations [9–12], and the current study was designed to examine T-cell activation in the context of the anticipated effect on leptin concentrations with calorie restriction, which was confirmed. Future work should look to design interventions and/or animal/ cell models to further establish the mechanistic link between these observations. Future studies may also wish to examine responses in different adipose tissue depots, and perhaps even different sub-locations within the same depot (e.g., deeper versus superficial).

## Conclusions

A reduction in leptin following short-term severe calorie restriction was temporally associated with a reduction in the number and levels of activation of blood CD4+ CD25+ T-lymphocytes. This reduction in T-lymphocyte activation in blood may be explained by energy intake being below requirements over this specific time period, and serum leptin is likely to play an important role in conveying this information to the immune system. This supports previous results seen in vitro. However, within subcutaneous adipose tissue, we did not observe a reduction in either leptin or T-lymphocyte activation. These between-compartment discrepancies may be due to differing responses of different tissues to calorie restriction and/or may depend on the specific mechanisms which explain the reduction in blood leptin. Collectively, these findings shed light on the link between leptin modulation and altered immune function during weight loss.

### Supplementary information

Supplementary Figure 1 and Table 1

## Data Availability

All data presented in this manuscript are available, upon request, from the University of Bath’s Data Repository (https://researchdata.bath.ac.uk/id/eprint/1350).
