# Supplementary material for: Calorie restriction-induced leptin reduction and T-lymphocyte activation in blood and adipose tissue in men with overweight and obesity
Source: Int J Obes (Lond). 2024 Mar 27;48(7):993–1002. doi: 10.1038/s41366-024-01513-7 (PMC11216992; doi:10.1038/s41366-024-01513-7)
Supplement: Supplementary file 1 — Supplementary Figure 1 and Table 1 [file 41366_2024_1513_MOESM1_ESM.pdf]

Supplemental Figure 1

Title: Impact of reduced leptin from caloric restriction on T-lymphocyte activation in blood and adipose in men with overweight and obesity

Supplementary information: Diet calculations for participants.

measured RMR	1761
TEE (actiheart)	3106
Avg energy In (diet records)	3123
Target daily calories:	1557.25

Target calories for each of the 3 days calculated as (TEE + Avg energy in)/4

1) Total energy intake (TEE) and Average energy intake entered and Target daily calories calculated.
2) Diet records entered into Excel with original weights consumed.
3) Conversion factor calculated for each diet record based on total calories in that diet option (3 options from participant's diet record selected for modification and they could choose from any of these during the 3 day 50 % calorie restriction period).
4) Weight of each item to be consumed based on target daily calorie intake (weight consumed in original diet x conversion factor).
5) Food/drink items and weights to be consumed entered into new diary for participants.

conversion =target / calories in option
copy and paste 0.50 below for all cells

Option 1

Day 1 calories	3100	0.50	1557.25
			Weight to be
food/drink	weight	conversion	consumed
Tesco gold coffee	4	0.50	2
sweetex x3		0.50	0
Hot water	252	0.50	127
skimmed milk	80	0.50	40
croissant	32	0.50	16
cafatiere coffee	240	0.50	121
sweetex x 3		0.50	0
skimmed milk	48	0.50	24
hovis white bread (toasted)	112	0.50	56
lurpak	28	0.50	14
marmite	4	0.50	2
coffee	250	0.50	126
skimmed milk	82	0.50	41
coffee	248	0.50	125
skimmed milk	80	0.50	40
mcvities chocolate digestives		0.50	4.5
carrot -raw	141	0.50	71
cafatiere coffee	270	0.50	136
skimmed milk	40	0.50	20
Etc...			

conversion=target / calories in option
copy and paste 0.51 below for all cells

Option 2

Day 2 calories	3073	0.51	1557.25
			Weight to be
food/drink	weight	conversion	consumed
cafatiere coffee	290	0.51	147
skimmed milk	40	0.51	20
sweetex x3		0.51	0
hovis white bread (toasted)	132	0.51	67
lurpak	12	0.51	6
marmite	7	0.51	4
cafatiere coffee	270	0.51	137
skimmed milk	52	0.51	26
raisins	15	0.51	8
sandwich bread	41	0.51	21
butter	5	0.51	3
marmite	3	0.51	2
cheddar cheese	49	0.51	25
cappacino	250	0.51	127
snackajack caramel (total during day)	75	0.51	38
twix	22	0.51	11
ham	108	0.51	55
fried eggs	118	0.51	60
bread	164	0.51	83
Etc...			

conversion=target / calories in option
copy and paste 0.41 below for all cells

Option 3

Day 3 calories	3799	0.41	1557.25
			Weight to be
food/drink	weight	conversion	consumed
coffee	290	0.41	119
skimmed milk	40	0.41	16
shreddies	82	0.41	34
skimmed milk	242	0.41	99
coffee	300	0.41	123
skimmed milk	50	0.41	20
celebrations	64	0.41	26
coke	500	0.41	205
cheddar	143	0.41	59
bread	202	0.41	83
onion	42	0.41	17
coffee	200	0.41	82
skimmed milk	40	0.41	16
tesco cherry lowfat yogurt	450	0.41	184
pear	240	0.41	98
grapes	50	0.41	20
special k bars (total during day)	75	0.41	31
bread	100	0.41	41
cheese	49	0.41	20
Etc...			

Supplementary Table 1. Overview of energy intake and total energy expenditure measured pre- and during the 3 day calorie restriction protocol. Energy expenditure was calculated by the sum of measured active energy expenditure and measured resting metabolic rate. Data are presented as mean ± SD. All data represent *n*=12.

**Average Energy Intake pre-intervention (kcal)**	**Prescribed Energy Intake during intervention (kcal)**	**Average TEE pre-intervention (kcal)**	**Average TEE during intervention (kcal)**
2499 ± 411	1319 ± 182	2781 ± 391	2734 ± 418

Abbreviations: TEE, total energy expenditure.
